# Ascitic Fluid/Serum Bilirubin Ratio as an aid in Preoperative Diagnosis of Choleperitoneum in a Neglected Case of Spontaneous Common Bile Duct Perforation

**DOI:** 10.5005/jp-journals-10018-1246

**Published:** 2017-09-29

**Authors:** Farhanul Huda, Manisha Naithani, Sudhir K Singh, Sarama Saha

**Affiliations:** 1 Department of General Surgery, All India Institute of Medical Sciences, Rishikesh, Uttarakhand, India; 2 Department of Biochemistry All India Institute of Medical Sciences, Rishikesh, Uttarakhand, India

**Keywords:** Biliary peritonitis, Choleperitoneum, Common bile duct, Spontaneous perforation.

## Abstract

Spontaneous perforation of extrahepatic biliary system is a rare and potentially fatal cause of acute abdomen. Clinical presentation is as biliary peritonitis. This condition is rarely suspected as a cause of peritonitis preoperatively and correct diagnosis is made during surgery. If suspected, diagnosis can be made by various imaging techniques like hepatobiliary scintigraphy, magnetic resonance cholangiopancreatography (MRCP), and endoscopic retrograde cholangiopancreatography (ERCP). As these imaging techniques are not readily available, especially in low socioeconomic countries, we hereby report a case of spontaneous common bile duct (CBD) perforation, which was diagnosed preoperatively by estimation of ascitic fluid-to-serum bilirubin ratio, a simple, quick, and cost-effective test.

**How to cite this article:** Huda F, Naithani M, Singh SK, Saha S. Ascitic Fluid/Serum Bilirubin Ratio as an aid in Preoperative Diagnosis of Choleperitoneum in a Neglected Case of Spontaneous Common Bile Duct Perforation. Euroasian J Hepato-Gastroenterol 2017;7(2):185-187.

## INTRODUCTION

Spontaneous perforation of the CBD is a rare disease in adults.^1^ Acute onset and delay in diagnosis lead to increased morbidity and sometimes mortality. Most of the cases are diagnosed intraoperatively because of their rarity. Preoperative diagnosis is made by hepatobiliary scintigraphy, MRCP, or ERCP, which are expensive and not readily available.^2,3^ We herein report a case of spontaneous CBD perforation that was diagnosed preoperatively by a simple and cost-effective test.

## CASE REPORT

A 40-year-old, chronic alcoholic male presented to our outpatient department with a history of on and off pain in the right upper abdomen since 3 months, and abdominal distension since 20 days. There was no history of vomiting, fever, diarrhea, yellowish discoloration of eyes or urine, upper or lower gastrointestinal bleeding. He was passing flatus and stools, and was taking a normal diet; however, his appetite had decreased. For these complaints, the patient was admitted in some other hospital where abdominal paracentesis was done twice, and 1,700 mL fluid was drained.

On examination, the patient was ill-looking, afebrile with tachycardia (102/minute), and normotensive (118/60 mm Hg). His body mass index was 19.0. Pallor and bilateral pitting edema were present. Abdominal examination revealed distension of abdomen with positive fluid thrill. Deep tenderness was present in right hypochondrium. There was no palpable lump. Liver dullness was not masked, and bowel sounds were normal. Baseline hematological investigations revealed leukocytosis, raised serum bilirubin (2.64 mg/ dL), alkaline phosphatase (283 U/L), gamma glutamyl transferase (148 U/L), and a low serum albumin (2.3 gm). Chest X-ray did not show any free gas under the right dome of diaphragm, and abdominal X-ray was normal. Ultrasound abdomen revealed gross ascites with cholelithiasis. Computed tomography (CT) or magnetic resonance imaging (MRI) abdomen could not be done as the patient was poor and could not afford it. Abdominal paracentesis revealed fluid with bilious tinge. In view of the confusing clinical scenario, the fluid was sent for bilirubin estimation. The ascitic fluid bilirubin was 21.17 mg/dL and the ascitic fluid:serum bilirubin ratio was 8.01.

**Fig. 1:**
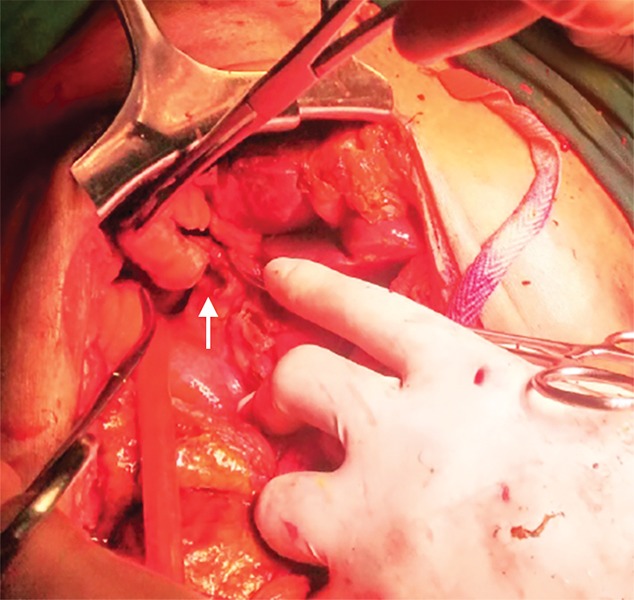
Perforation in CBD

With the finding of cholelithiasis, bile-tinged ascites, and the ascitic fluid/serum bilirubin ratio of 8.01, a preoperative diagnosis of spontaneous perforation of extrahepatic biliary tree was made and the patient was taken up for exploratory laparotomy. On exploration, gross biliary ascites (2.5 L) was found. Thin, flimsy adhesions and bilious fluid were present in between the gut loops along with edematous omentum. Stomach, small and large bowels were grossly normal. Gallbladder was distended and had thickened wall with an impacted stone of size 2 cm at the neck. A perforation of size 0.5 × 0.5 cm was found over the anterolateral wall of the CBD, just below the junction of common hepatic duct and cystic duct (Fig. 1). The CBD perforation had unhealthy margins and was constantly oozing bile. The CBD exploration through same perforation revealed no evidence of distal obstruction. Thorough peritoneal lavage was done and repair of CBD perforation was done over a T-tube introduced through the perforation. Cholecystectomy was performed. Postoperative recovery of the patient was well. He was allowed oral intake on third postoperative day. A T-tube cholangiogram was done on the 14th day after surgery, which showed a small peritubular leak, but there was no filling defect. The patient was sent home on the T-tube, which was removed on the next follow-up after 7 days.

## DISCUSSION

Spontaneous perforation of CBD is very rare in adults. It was first described by Freeland in 1882. Since then, very few cases have been reported in the literature.^4^ The causes for spontaneous CBD perforation reported in the literature are increased intraductal pressure due to stone, tumor, stricture, erosion of a biliary diverticulum, or post-ERCP.^5^ The commonest presentation is insidious onset of progressive jaundice and abdominal distension.^6^ Owing to its rarity, the diagnosis of spontaneous CBD perforation is usually made peroperatively.^3^ A preoperative diagnosis can be made with the help of cholescintigraphy, MRI, ERCP, or CT. But these tests are costly and not readily available.

Normal range of ascites fluid bilirubin in patients is 0.7 to 0.8 mg/dL. Ascitic fluid bilirubin concentration of >6 mg/dL supports the diagnosis of choleperitoneum.^7^ The study done by Darwin et al^8^ showed that drain fluid to serum bilirubin ratio >5 is highly sensitive and specific of bile leak. DeBenedet et al^9^ reported that ascitic fluid/serum bilirubin ratio of >3.25 produced optimal specificity and sensitivity to identify bile leaks in orthotopic liver transplant recipients. In limited resource settings, these simple and cost-effective biochemical tests can be used in the preoperative diagnosis of choleperitoneum. In our case, we were not sure regarding the cause of ascites as the patient was not icteric and diagnosis of choleperitoneum was made based on the biochemical test of acsitic fluid.

The recommended treatment of this entity is cho-lecystectomy with T-tube drainage of CBD.^10^ A chole-cystostomy can be done if there are adhesions and the anatomy is not clear. If perforation is large, gallbladder flap may be applied for repair of perforation; and Roux-en-Y hepaticojejunostomy is the procedure of choice if there is complete ductal disruption. Primary repair of CBD is considered hazardous due to local inflammation.

## CONCLUSION

Spontaneous CBD perforation is a rare but fatal condition in adults. Awareness of this condition as a rare cause of choleperitoneum avoids undue delay in diagnosis and improves outcome. We recommend estimation of ascitic fluid bilirubin level and ascitic fluid-to-serum bilirubin ratio to diagnose choleperitoneum preoperatively, especially in doubtful diagnosis, as a simple, quick, and cost-effective test. Early surgical intervention helps to prevent morbidity and mortality.
